# Molecular Assessment of Domain I of Apical Membrane Antigen I Gene in *Plasmodium falciparum*: Implications in *Plasmodium* Invasion, Taxonomy, Vaccine Development, and Drug Discovery

**DOI:** 10.1155/2022/1419998

**Published:** 2022-10-07

**Authors:** Che Roland Achungu, Damian Nota Anong, Robert Adamu Shey, Cevie Jesenta Tabe

**Affiliations:** ^1^Department of Microbiology and Parasitology, Faculty of Science, University of Buea, Buea, Cameroon; ^2^Laboratory of Molecular Parasitology, University of Buea, Buea, Cameroon; ^3^Florence Nightingale Higher Institute of Health and Biomedical Sciences, Bamenda, Cameroon; ^4^Department of Biological Sciences, The University of Bamenda, Bamenda, Cameroon; ^5^Department of Biochemistry and Molecular Biology, Faculty of Science, University of Buea, Buea, Cameroon

## Abstract

Given its global morbidity and mortality rates, malaria continues to be a major public health concern. Despite significant progress in the fight against malaria, efforts to control and eradicate the disease globally are in jeopardy due to lack of a universal vaccine. The conserved short peptide sequences found in Domain I of *Plasmodium falciparum* apical membrane antigen 1 (PfAMA1), which are exposed on the parasite cell surface and in charge of *Plasmodium falciparum* invasion of host cells, make PfAMA1 a promising vaccine candidate antigen. The precise amino acids that make up these conserved short peptides are still unknown, and it is still difficult to pinpoint the molecular processes by which PfAMA1 interacts with the human host cell during invasion. The creation of a universal malaria vaccine based on the AMA1 antigen is challenging due to these knowledge limitations. This study used genome mining techniques to look for these particular short peptides in PfAMA1. Thirty individuals with *Plasmodium falciparum* malaria had blood samples taken using Whatman's filter papers. DNA from the parasite was taken out using the Chelex technique. Domain I of the *Plasmodium falciparum* AMA1 gene was amplified using nested polymerase chain reactions, and the amplified products were removed, purified, and sequenced. The DNA sequence generated was converted into the matching amino acid sequence using bioinformatic techniques. These amino acid sequences were utilized to search for antigenic epitopes, therapeutic targets, and conserved short peptides in Domain I of PfAMA1. The results of this investigation shed important light on the molecular mechanisms behind *Plasmodium* invasion of host cells, a potential PfAMA1 vaccine antigen sequence, and prospective malaria treatment options in the future. Our work offers fresh information on malaria medication and vaccine research that has not been previously discussed.

## 1. Introduction

Given its high global morbidity and mortality rates, malaria continues to be a major public health concern. Malaria is a parasite disease spread by mosquitoes. *Plasmodium falciparum*, *Plasmodium vivax*, *Plasmodium malariae*, *Plasmodium ovale*, and *Plasmodium knowlesi* are the five protozoa that cause malaria in humans [1]. *Plasmodium vivax* is the leading cause worldwide, whereas *Plasmodium falciparum*, which causes more than 90% of global malaria death, continues to be the single most significant hazard to public health on a global level [1]. Malaria often causes severe disease, including high fever, shivering chills, and flu-like symptoms. The predicted number of malaria cases and deaths globally in 2020 was 241 million and 627,000, respectively [2]. With the development of antimalarial medications like artemisinin, the implementation of efficient vector control strategies, and, most recently, the WHO's approval of RTS,S/AS01 vaccine to be used as the first malaria vaccine for children under five years of age [2], with 39% efficacy against malaria [3], control efforts have produced significant gains in the fight against malaria globally. However, the development and potential spread of artemisinin resistance in the malaria parasite, pesticide resistance in the mosquito vector, and the insufficient protection provided by the RTS,S/AS01 vaccine put these accomplishments in jeopardy. These characteristics obstruct attempts to control and eradicate malaria globally, underscoring the critical need for the creation of malaria vaccines that are both highly effective and secure.

The majority of the malaria vaccines that have undergone field trials have demonstrated modest efficacy. Polymorphisms in the genes that code for the vaccine candidate antigens could be one cause of the low efficacy. Apical membrane antigen 1 (AMA1) is a significant vaccine candidate antigen. AMA1 is an integral membrane protein with a 55-amino acid cytoplasmic segment and a 550-amino acid extracellular region that can be divided into three domains (Domains I, II, and III) based on intradomain disulphite bonds [4]. AMA1 is expressed in the late schizont stage of the parasite and necessary for merozoites to invade erythrocytes and sporozoites to invade hepatocytes [5], despite the fact that its role is still poorly understood. It has been demonstrated that antibodies against AMA1 prevent parasite invasion of human erythrocytes [6]. Because AMA1 is involved in both the invasion of hepatocytes by sporozoites and the invasion of erythrocytes by merozoites, it offers a special possibility as a candidate antigen for a multistage vaccine. The polymorphisms found in the AMA1 gene [7] and the lack of precise knowledge of the molecular mechanisms governing AMA1 interaction with host cells have made it challenging to develop an effective malaria vaccine over time. The majority of the single-nucleotide polymorphisms (SNPs) have been shown to be present in the highly immunogenic Domain I of AMA1 [8]. Additionally, RON2 has a binding site in Domain I of AMA1, which is necessary for the creation of actin-myosin-associated moving junctions, which are crucial for parasite invasion of host cells. A conserved area of Domain I of AMA1 that is surrounded by highly polymorphic regions is where RON2 binds [9]. It is yet unknown which short peptide sequences make up this conserved region of Domain I of AMA1 that interacts with RON2. Furthermore, it is still unclear how AMA1 interacts with host cells during parasite invasion through molecular pathways. These knowledge gaps hamper the creation of medicines and vaccines that target AMA1. This study sought to determine whether the conserved short peptide sequences in Domain I of PfAMA1 that bind to RON2 could be helpful for the production of vaccines and the discovery of new medicines.

## 2. Methods

### 2.1. Study Area

The research was done at Bamenda. The capital of Cameroon's Northwest Region, Bamenda, is located between latitude 6 N and longitude 10.1 E. 1472 meters above the sea level is the average altitude. The climate is classified as a tropical monsoon with two seasons: dry season from November to March and rainy season from April to October. The annual rainfall is 2300 mm. Bamenda, which has a population of about 900000, is 366 kilometers northwest of Yaounde, the capital of Cameroon. Because it is an urban region with individuals from several ethnic groups, its population is genetically varied. The spread of malaria is ongoing. The frequency of malaria rises in the wet season and declines in the dry season. From June through August 2019, blood samples were taken from participants in the study.

### 2.2. Sample Collection and Processing

Venous blood samples were taken from 15 boys and 15 girls aged 0 to 5 years who are all from the semi-Bantu ethnic group with no history of immune suppression diseases and displayed malarial symptoms and clinical indications. A total of 30 blood samples were collected. The blood samples were subjected to rapid diagnostic tests and microscopic examination through thick and thin blood films. Blood samples positive for *Plasmodium falciparum* were spotted on Whatman filter papers packaged individually in a zip-locked bags and appropriately labeled. The samples were examined at the University of Buea's Molecular Parasitology Laboratory.

### 2.3. Malaria Parasite DNA Extraction

*Plasmodium falciparum* genomic DNA was extracted using the Chelex method of DNA extraction as described previously [10]. Agarose gel electrophoresis was used to confirm the existence of genomic DNA. The extracted DNA was kept at −20°C until it was needed.

### 2.4. Genotyping

This was done using the nested PCR approach as described previously [7].

### 2.5. Primary PCR

The PCRs were carried out on a Gene AMP® PCR 9700 Applied Biosystem Machine. The PCR mixture contained 10 *µ*l of OneTaq Hot Start Quick-Load (from New England Biolabs), 0.8 *µ*l of forward primers, 0.8 *µ*l of reverse primers, 1 *µ*l of DNA template from the extracted DNA, and 7.4 *µ*l of nuclease-free water making a total volume of 20 *µ*l. The primers used amplified the complete PfAMA1 gene. The PCR was programmed for 25 cycles.

### 2.6. Secondary PCR

The PCRs were carried out on a Gene AMP® PCR 9700 Applied Biosystem Machine. The PCR mixture contained 10 *µ*l of OneTaq Hot Start Quick-Load (from New England Biolabs), 0.8 *µ*l of forward primers, 0.8 *µ*l of reverse primers, 1 *µ*l of DNA template obtained from the product of the first round of PCR, and 7.4 *µ*l of nuclease-free water making a total volume of 20 *µ*l. The primers used amplified Domain I of PfAMA1 where majority of genetic diversity occurred. The PCR was programmed for 30 cycles. Cycling conditions for both PCRs were as follows: initial denaturation at 95°C for 5 minutes, followed by 25 cycles (first round) or 30 cycles (second round) of denaturation at 94°C for 1 minute, annealing at 58°C for 2 minutes, and extension at 72°C for 2 minutes. The final extension was carried out at 72°C for 5 minutes and held at 5°C. The negative control's PCR settings were the same as those used in the experiment, but it did not contain any of the genomic DNA that was extracted from *Plasmodium falciparum*. The primers used are shown in Table 1.

### 2.7. Visualizing AMA1 PCR Products Using Agarose Electrophoresis

**Figure 1:**
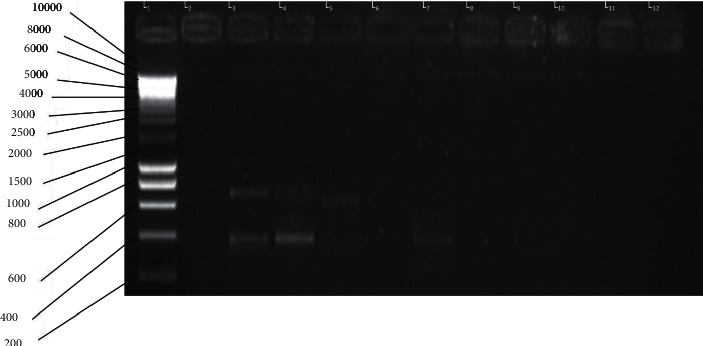
Exemplary gel showing AMA1 fragments produced after the second round of PCR (created by the authors of this work). L_1_: molecular weight marker (10,000 bp); L_3_: variants with 700 bp and 400 bp; L_4_: variant with 400 bp; L_5_: variant with 700 bp; L_7_: variant with 400 bp; L_12_: negative control.

The amplified products of the secondary PCR were visualized using 1.5% agarose gel by electrophoresis. The agarose gel contained 1.5% agarose mixed with 1*µ* of ethidium bromide. Electrophoresis ran for 40 minutes at 100 V. The migration occurred in a gel filled with 1X TBA buffer. A molecular weight marker (10000 bp) was loaded in the first well. The migrated DNA products were visualized using the gel documentation system (Bio-Rad, USA). The migrated amplified DNA fragments are shown in Figure 1.

Two major bands were identified: one approximately of 700 bp and the other approximately of 450 bp. The two bands were cut and removed from lane 3 (L_3_) of the gel. The bands were purified and sequenced on a BigDye V3.1 sequence machine using the forward primer GGAACTCAATATAGACTTCC and analysed on an ABI 3130XL Genetic Analyzer.

### 2.8. Analysis

The *Plasmodium falciparum* apical membrane antigen 1 (AMA1) nucleotide sequence data were arranged using the BioEdit software tool. NCBI-BLAST (National Center for Biotechnology Information-Basic Local Alignment Search Tool) was used to find regions of local similarity to the *Plasmodium falciparum* 3D7 reference genome and other related sequences in the NCBI database. The protein coding sequence (CDS) was identified in the sequence using BLAST-CDS. The 3D structure of the protein obtained was predicted using the GalaxyWEB tool, and the ligand binding sites were predicted using the 3DLigandSite tool. Similar conserved domain architectures were determined using NCBI-BLAST Conserved Domain Architecture Retrieval Tool. Immune Epitope Database (IEDB) and Support Vector Machine Tri-peptide (SVMTrip) software tools were used to predict antigenic epitopes from the protein sequence of Domain I of PfAMA1 obtained in the study. The VaxiJen V2.0 bioinformatic tool was used to check for immunogenic properties of the protein sequence of Domain I of PfAMA1 obtained in the study.

## 3. Results

### 3.1. Gene Sequence Similarities of Domain I of *Plasmodium falciparum* AMA1

In order to investigate gene sequence similarities of Domain I of *Plasmodium falciparum* AMA1 obtained in the study, the bioinformatic tool NCBI-BLAST was used. The 459 bp sequence produced showed high significant similarities to *Plasmodium falciparum* AMA1 gene sequences found in the NCBI database. It showed 96.38% identity with 96% query cover with the *Plasmodium falciparum* 3D7 reference genome (Accession number: NC037282.1) and 99.57% identity after 96% query cover with the *Plasmodium falciparum* apical membrane antigen 1 (AMA1) gene partial CDS of 100 selected sequences in the NCBI database. The 459 bp sequence was deposited into the GenBank database and was assigned a genome (Accession number: OL634842).

### 3.2. Identification of Conserved Short Peptides in Domain I of *Plasmodium falciparum* AMA1

**Figure 2:**
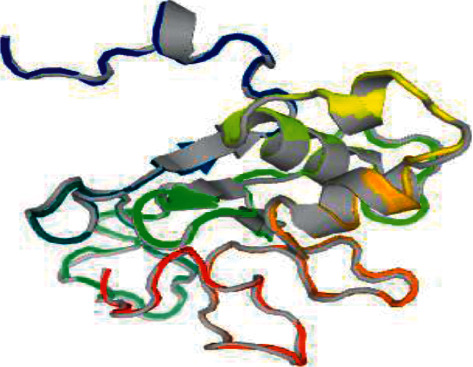
3D protein structural prediction of the 149-amino acid PfAMA1 protein sequence obtained in the study (using galaxy.seoklab.org).

**Figure 3:**
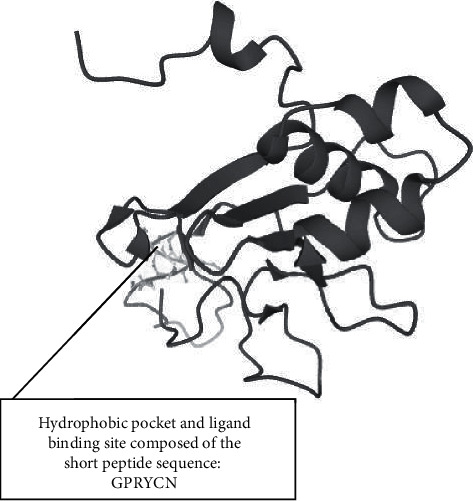
Predicted ligand binding sites [11].

In order to determine the conserved short peptides in Domain I of *Plasmodium falciparum* AMA1 that could be potential drug targets and the hydrophobic pocket of AMA1, the 459 bp gene sequence was translated into its corresponding protein sequence (Figure. S1) using a bioinformatic tool. The protein sequence was used in protein-to-protein BLAST, using the bioinformatic tool NCBI-BLAST to obtain similar protein sequences of Domain I of *Plasmodium* AMA1 in all the human *Plasmodium* species (Figure. S2). The short peptide sequences are conserved across *Plasmodium* species surrounded by a highly polymorphic region [9]. To investigate this, the protein sequence obtained (Figure. S1) was used to compare with all the different protein sequences (Figure. S2) using the bioinformatic tool EMBOSS Needle. The short peptide sequences PAVYD and GPRYC are conserved across the entire *Plasmodium* species infecting human (Table 2). To test whether these conserved short peptide sequences might be the hydrophobic pocket of *Plasmodium* AMA1, the 3D structure of the protein sequence obtained in the study was predicted using a bioinformatic tool (Figure 2). To identify the short peptide sequences that make up the hydrophobic pocket and ligand binding site in the 3D structure of the protein, the bioinformatic tool developed by Wass et al. [11] was used. The short peptide GPRYC was predicted as part of the hydrophobic pocket and ligand binding site (Figure 3). In addition, to predict which of the amino acid residues in the short peptide sequence (GPRYC) might be involved in ligand binding, the bioinformatic tool developed by Wass et al. [11] was used. Cysteine (C) and proline (P) were predicted as more likely to bind to the ligand and interact with it (Table 3).

### 3.3. Immunogenic Properties of Domain I of *Plasmodium falciparum* AMA1

To determine the immunogenic properties of the protein sequence of Domain I of *Plasmodium falciparum* AMA1 obtained in the study, the bioinformatic tools IEDB and SVMTrip were used to predict linear B-cell epitopes in the protein sequence. The IEDB tool predicted six B-cell epitopes in the sequence (Table 4). The SVMTrip tool predicted six B-cell epitopes but recommended two as indicated by the flags (Table 5).

Common West African HLA antigens associated with protection against severe malaria: HLA-B53, HLA-DQB1 ^*∗*^05:01, and HLA-DRB1 ^*∗*^13:02 [12], were used for T-cell epitope prediction. The sequence shows that it is a good binder for HLA-B53 (the MHC class I binding predictions were made on 11/7/2021 using the IEDB analysis resource NetMHCpan [13]). For MHC class II, the sequence indicates that it is a good binder for HLA-DQB1 ^*∗*^05:01 and HLA-DRB1 ^*∗*^13:02 (the MHC class II predictions were made on 11/7/2021 using the IEDB analysis resource consensus tool [14]). The results from IEDB analyses also indicate that the protein sequence is more likely to be processed naturally by MHC molecules.

To investigate whether the protein sequence obtained in the study is an antigen and an immunogen (protective antigen), the bioinformatic tool VaxiJen V2.0 was used to predict the protective antigen and subunit vaccine sequence. For the parasite antigen, the protective antigen is predicted if the value obtained is greater than 0.5. The result obtained shows that the sequence is more likely to be a protective antigen as the predicted value is 0.5436 (Figure. S3). In addition, to determine whether the protein sequence obtained in the study is conserved across *Plasmodium falciparum*, protein-to-protein BLAST using the bioinformatic tool NCBI-BLAST was performed. The result obtained indicates that the protein sequence is conserved across *Plasmodium falciparum*.

### 3.4. Predicted Binding Ligands

**Figure 4:**
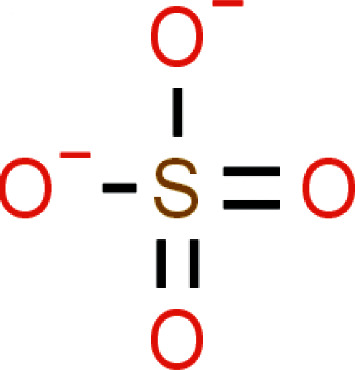
Sulphate ion (Drug identity: DB14546) bound to the peptide sequence GPRYCN.

**Figure 5:**
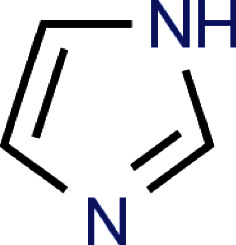
Imidazole (Drug identity: DB03366) bound to the short peptide NL.

To identify possible future drugs against malaria, the bioinformatic tool developed by Wass et al. [11] was used to identify ligands that can bind to the short peptide sequence GPRYC and to other areas of the 3D structure of the protein obtained in the study. The sulphate ion (Figure 4) was identified as a potential ligand that can bind to the short peptide sequence GPRYCN. Imidazole (Figure 5) was identified as a potential ligand that can bind to the short peptide sequence NL found in the protein 3D structure.

### 3.5. Taxonomy

To determine how the human *Plasmodium* species are related based on Domain I of *Plasmodium* AMA1, the protein sequence obtained in the study (Figure. S1) and those obtained in the NCBI database (Figure. S2) were used in a pairwise comparison using the bioinformatic tool EMBOSS Needle. The result indicates that *Plasmodium vivax* and *Plasmodium knowlesi* are closely related (83%) identity in their amino acid sequences found in their Domain I of *Plasmodium* AMA1 (Table 6). Pairwise comparison between the major human *Plasmodium* species and some major *Plasmodium* species indicates that *Plasmodium falciparum* and *Plasmodium reichenowi* are closely related (91.3%) identity in their amino acid sequences found in their Domain I of AMA1. *Plasmodium vivax* and *Plasmodium cynomolgi* are closely related (89.3%) identity in their amino acid sequences found in their Domain I of AMA1 (Table. S1).

### 3.6. Similar Conserved Domain Architectures

To predict the function of Domain I of *Plasmodium falciparum* AMA1 obtained in the study, similar conserved domain architectures were obtained from the NCBI database using the protein sequence obtained in the study. These similar conserved domains are apical membrane antigen 1 partial, somatic embryogenesis receptor kinases, and leucocyte tyrosine kinase receptor. Domains are evolutionary conserved units of proteins, are widely used to classify protein sequences, and infer protein functions [15]. So based on these, we hypothesize that Domain I of *Plasmodium falciparum* AMA1 might be an extracellular receptor tyrosine kinase where the receptor pocket might be composed of the short peptide sequences PAVYD and GPRYC.

## 4. Discussion

### 4.1. Proposed Molecular Mechanisms for *Plasmodium* Invasion of Host Cells

*Plasmodium* uses apical secretory organelles to invade host cells. The invasion of host cells begins with the proteins secreted from the micronemes of the parasite and targeted to the parasite surface where they engage with host cell receptors [16]. This process triggers subsequent secretion of Rhoptry neck proteins from the secretory organelle of the parasite called the rhoptries. These Rhoptry neck proteins consist of RON2, RON4, RON5, and RON8 which are exported to the host cell surface membrane. RON2 is integrated into the host cell surface membrane with its N-terminal domain directed into the cytosol where it is likely retaining RONs 4, 5, and 8 to the cytosol face of the host membrane [17]. RON2 serves as a ligand for micronemal-secretory apical membrane antigen 1 (AMA1) secreted from the microneme and exported to the parasite cell membrane shortly before invasion. AMA1 interacts directly with the extracellular C-terminal portion of RON2 [18], resulting in the formation of the moving junction (MJ). The formation of MJ is considered the irreversible attachment step necessary for successful invasion [19]. The parasite then actively passes through the MJ apparently using an actin-myosin motor [20]. This results in the internalization of the parasite into a parasitophorous vacuole created by the host cell. During MJ formation, RON2 adopts a conformation that enables it to span the host cell membrane such that a disulphide-constrained loop near its C-terminus can interact with AMA1 [21]. Interfering with this RON2-AMA1 interactions blocks parasite invasion of the host cell [22]. However, the molecular processes that lead to the creation of the moving junction and the parasitophorous vacuole are still unclear, as are the peptide sequences of AMA1 that generate the hydrophobic pocket for the RON2 ligand.

The results of this study indicate that the *Plasmodium* AMA1 hydrophobic pocket might be composed of the short peptide sequences GPRYC and PAVYD. One side of the hydrophobic pocket might consist of the short peptide sequence GPRYC. The other side of the hydrophobic pocket might consist of the short peptide sequence PAVYD. G is opposite P, P is opposite A, R is opposite V, Y is opposite Y, and C is opposite D. The short peptide sequence (GPRYC) on one side of the pocket begins with glycine (G) from the inside of the pocket to cysteine (C) on the outside of the pocket. In contrast, on the other side of the pocket, the short peptide sequence (PAVYD) begins from proline (P) from the inside of the pocket to aspartic acid (D) on the outside of the pocket. Glycine (G) at the hydrophobic pocket permits any combination of side chains which otherwise would have to be excluded because of steric hindrance. For this reason, glycine provides a means for the juxtaposition of the two tyrosine residues in the pocket. Proline (P) plays a role in the formation of the hydrophobic pocket by changing the direction of the polypeptide chains. Proline may also facilitate sequence-specific recognition of RON2 protein by AMA1 without requiring a particular high affinity interaction and facilitate binding of RON2 to AMA1. Prior to RON2 binding, the two tyrosine residues in the pocket might play a role in molecular recognition. This molecular recognition enables AMA1 to identify RON2 in the midst of other host cell membrane surface proteins. Arginine (R) in position 103 and cysteine (C) in position 105 might be involved in the binding of AMA1 to RON2. The previous work [23] has shown that RON2 has a conserved aspartic acid (D) and cysteine (C) that are critical for AMA1 binding to RON2. Based on this, we suggest that the conserved cysteine on AMA1 forms an interchain disulphide bridge with the conserved cysteine in RON2. Since arginine is a positive amino acid and aspartic acid is a negative amino acid, the conserved arginine on AMA1 forms an ionic bond with the conserved aspartic acid on RON2. So, RON2 might be attached to AMA1 with the help of an interchain disulphide bridge and an ionic bond. Since the N-terminal of RON2 is integrated in the host cell membrane, these bonding connections provide a firm attachment of the parasite to the host cell during the gliding movement that ends with the parasitophorous vacuole formation.

The results of this study also suggest that Domain I of *Plasmodium* might be a receptor kinase. The aspartic acid residue (D) on PAVYD might be involved in the regulation of enzymatic activity and the expression of receptor tyrosine kinase after the binding of RON2 to the AMA1 hydrophobic pocket. Valine on PAVYD might promote the activation of tyrosine kinases in the pocket, and this view is supported by the work of Dimaio and Irusta [24]. So based on these, we suggest that the AMA1 hydrophobic pocket might be an extracellular tyrosine kinase-like receptor where binding of RON2 to the pocket activates the kinase activity in the pocket. This activation gives rise to cross phosphorylation of the juxtaposed tyrosine residues (Y) on GPRYC and PAVYD by kinases. This phosphorylation might generate molecular signals that are transmitted to the actin-myosin motor in the cytoplasm of the parasite with the help of the cytoplasmic tail of AMA1. This motor after receiving the signals starts the gliding movement that enables the parasite to move into the pore formed on the host cell membrane resulting in the parasitophorous vacuole. All these claims are totally hypothetical and needed to be confirmed by assays.

The molecular mechanisms by which *Plasmodium* species interact with human host cells during cell invasion have been difficult to explain previously. The lack of this knowledge previously makes the development of a universal malaria vaccine based on AMA1 antigen difficult and also reduces the prospect for the discovery of new antimalarial drugs that target AMA1. This study suggested for the first time the molecular mechanisms by which *Plasmodium* species invade human host cells and also identified the short peptides GPRYC and PAVYD to be crucial for *Plasmodium* invasion of human host cells. We suggest that smaller ligands or antibodies that bind to GPRYC might block *Plasmodium* invasion of human host cells. Future studies will be necessary to validate this apparent suggestion which will open a new research for the discovery of new drugs and vaccines against malaria.

### 4.2. Vaccine Development and Drug Discovery

The protein sequence (Figure. S1) obtained in the study shows no significant similarity to any human protein in the NCBI database. This makes the sequence a good vaccine antigen because when antibodies are produced against the antigen in the human body, they cannot attack the body's own antigens because of the great diversity. The sequence may contain B-cell epitopes based on the linear B-cell epitopes predicted from the sequence (Tables 3 and 4). The sequence may be processed by body cells and then presented to CD4 and CD8 cells utilizing MHC class II and MHC class I molecules, respectively, according to T-cell epitope predictions. The sequence is an antigen, as evidenced by this. The sequence was further tested for immunogenic properties. The result indicates that the sequence may be an immunogen (Figure. S3). Protective antigens (immunogens) are specifically targeted by the acquired immune response of the host and are able to induce protection in the host against infectious and noninfectious diseases [25]. Protective antigens play important roles as biological markers for disease diagnosis, vaccine development, and analysis of fundamental host immunity against diseases [25]. Most *Plasmodium falciparum* antigens that have been used for vaccine studies are highly immunogenic showing good promise as vaccine candidates, but most of these antigens are also highly polymorphic and elicit parasite strain-specific immune responses. Over the years, it has been difficult to produce an effective vaccine against malaria using AMA1 because of polymorphisms observed in the AMA1 gene [7]. This study provides a protein sequence of Domain I of *Plasmodium falciparum* AMA1 that has immunogenic properties and is conserved across *Plasmodium falciparum*. This protein sequence may serve as a potential vaccine sequence in the future. The protein sequence (Supplementary Figure 1) shows significant similarities to the AMA1 sequences that were used in AdCh63 AMA1, DNA-Ad, and PfAMA1 vaccines that are currently being tested in clinical trials. This protein sequence obtained from this study provides a useful alternative sequence if the clinical trials of the above-mentioned vaccines face difficulties with respect to efficacy.

One of the main obstacles to the global control and eradication of malaria is the establishment and potential spread of artemisinin resistance by human *Plasmodium* species, underscoring the urgent need for the creation of new antimalarial medications. This investigation identified imidazole (Figure 5) and sulphate ion (Figure 4) as two potential future medications for the treatment of malaria. Imidazoles are antifungi drugs that inhibit the synthesis of ergosterol in fungi [26]. Imidazoles have been reported in previous works of having in vitro activities against *Plasmodium falciparum* [27, 28]. There have been different reports on the mode of actions of imidazoles on *Plasmodium falciparum*, suggesting that imidazoles may act on a variety of targets on *Plasmodium falciparum*. This study for the first time identified a possible target of imidazole on Domain I of *Plasmodium falciparum* AMA1 and also supported the view that imidazoles such as clotrimazole and ketoconazole may in the future be considered possible drugs against malaria.

The sulphate ion (Figure 4) drug is still under development, and the protein target for the drug is not yet known. This drug has been used in clinical trials against respiratory diseases (ClinicalTrials.gov Identifier: NCT02084043). This study identified Domain I of *Plasmodium falciparum* AMA1 as a possible protein target for this drug. This study indicates that this medication should be investigated as a potential malaria treatment in the future. However, in order for this to happen, additional in vitro research will be required to support this apparent recommendation.

### 4.3. Taxonomy

Protein sequence analysis of Domain I of human *Plasmodium* AMA1 reveals a close relationship between *Plasmodium vivax* and *Plasmodium knowlesi*. This might explain why these particular human *Plasmodium* species are the only ones that can bind to the erythrocytes' Duffy antigen receptor for chemokines. *Plasmodium malariae* and *Plasmodium ovale* are closely related to *Plasmodium vivax* than *Plasmodium falciparum* (Table 6). Comparing the protein sequence of Domain I of AMA1 in the major human *Plasmodium* species (*Plasmodium vivax* and *Plasmodium falciparum*) with the protein sequence of Domain I of AMA1 in other major *Plasmodium* species (*Plasmodium berghei*, *Plasmodium cynomolgi*, and *Plasmodium reichenowi*) indicates that *Plasmodium vivax* is closely related to *Plasmodium cynomolgi* (89.3% identity). *Plasmodium vivax*, *Plasmodium knowlesi*, and *Plasmodium cynomolgi* might have originated from a common ancestor. These findings are similar to those obtained by Tachibana et al. [29]. *Plasmodium falciparum* is closely related to *Plasmodium reichenowi* (91.3% identity) than to any of the human *Plasmodium* species suggesting a common ancestor for these two *Plasmodium* species. The result from this study indicates that amino acid sequences of Domain I of human *Plasmodium* AMA1 differ across human *Plasmodium* species.

Even though differences exist among amino acid sequences of Domain I of human *Plasmodium* AMA1, the short peptide sequences PAVYD and GPRYC remain conserved across amino acid sequences of Domain I of human *Plasmodium* AMA1 (Table 2). A study has shown that they appear to be a significant conservation of the invasion apparatus on the level of both ultrastructure and protein associated with apical organelles [30]. These common elements are of particular interest biologically as they constitute the phylogenetic conserved basic machinery for host cell invasion essential for the survival of these obligate intracellular parasites [30]. Single-nucleotide polymorphisms (SNPs) occur in the AMA1 gene across apicomplexan species because AMA1 is a target for host immunity. These polymorphisms might occur as a result of pressure exerted on AMA1 by the host immune system [31]. These polymorphisms might be immune evasion polymorphisms. Despite these polymorphisms, the invasion ligand that fills the hydrophobic pocket of AMA1 is functionally conserved throughout apicomplexan species. This invasion ligand binds to a conserved sequence of amino acid in RON2 [32]. A study has demonstrated that genetic engineered *Plasmodium falciparum* having *Plasmodium vivax* AMA1 was able to bind to *Plasmodium falciparum* RON2 and invade host cells generating new chimeric *Plasmodium falciparum* [6]. This functional conservation of invasion ligand happens to occur across species of apicomplexans but not among genera as *Plasmodium falciparum* RON2 failed to bind to *Toxoplasma* AMA1 [22]. Thus, the AMA1-RON2 binding is evolutionarily conserved across apicomplexan species. An in vitro study has demonstrated that induced mutation in one of the amino acids in the hydrophobic pocket prevents the binding of AMA1 to a complex of RON2 proteins [33]. The short peptide sequences PAVYD and GPRYC may constitute the conserved basic machinery for human host cell invasion by human *Plasmodium* species.

Each apicomplexan species utilized its own parasite-derived RON2 protein as a receptor rather than a conserved host molecule. Sequence polymorphisms occur in RON2 protein across apicomplexan genera [22] and across apicomplexan species [34]. These single-nucleotide polymorphisms might be immune evasion polymorphisms as RON2 is a target for host immunity [34]. Although RON2 protein has variations in different apicomplexan species, the peptide sequence of RON2 protein that binds to the AMA1 hydrophobic pocket is conserved across species of apicomplexans [32, 35].

## 5. Conclusion

In this investigation, the short peptides GPRYC and PAVYD that are conserved throughout *Plasmodium* AMA1 Domain I were discovered. It is possible that GPRYC and PAVYD make up *Plasmodium* AMA1's hydrophobic pocket. Additionally, the findings imply that the hydrophobic pocket of *Plasmodium* AMA1 may represent an external receptor tyrosine kinase. This research also sheds important light on a prospective malaria vaccine antigen sequence, potential future malaria drugs, and molecular mechanisms of *Plasmodium* penetration of host cells. Our research sheds light on malaria treatment and vaccine development in a way that has not been previously discussed.

## Figures and Tables

**Table 1:** Primers used to genotype the *Plasmodium falciparum* AMA1 gene.

Target	Primers	Sequence 5′-3′
Whole PfAMA1 gene First round of PCR	Forward	CCGGATCCCCTTTGAGTTTACATATATG
	Reverse	AAATTCTTTCTAGGGCAAAC
Domain I of PfAMA1 Second round of PCR	Forward	GGAACTCAATATAGACTTCC
	Reverse	AAATTCTTTCTAGGGCAAAC

**Table 2:** Similar conserved amino acid sequences in Domain I of AMA1 in *Plasmodium* species that infect human.

Amino acid positions	Pf	Pv	Pm	Po	Pk
7–13	FLKPVAT	FLKPVAT	FLTPVAT	FLKPVAT	FLTPVAT
18–26	LKDGGFAFP	LKDGGFAFP	LKSGGFAFP	LKSGGFAFP	LKEGGFAFP
33–36	SPMT	SPMT	SPVT	SPIS	SPIT
44–47	YKDN	YKDN	YEEH	YNEN	YKEN
53–59	LDELTLC	LNDIALC	LNDLSLC	LNDMSLC	LNDIALC
79–83	PAVYD	PAVYD	PAVYD	PAVYD	PAVYD
101–105	GPRYC	GPRYC	GPRYC	GPRYC	GPRYC
131–136	YLSKNV	YLSKNV	YLSKNV	YLSKNL	YLSKNV
144–149	CPRKNL	CPRKNL	CPRKSL	CPRNNL	CPRKNL

Pf = *Plasmodium falciparum*; Pv = *Plasmodium vivax*; Pm = *Plasmodium malariae*; Po = *Plasmodium ovale*; Pk = *Plasmodium knowlesi*.

**Table 3:** Predicted binding residues that interact with the ligand [11].

Residue	Amino acid	Conservation (0 and 1)	Solvent accessibility	Probability (0 and 1)
101	GLY	0.0	35.32	0.99
102	PRO	0.0	112.09	1.0
103	ARG	0.0	202.44	0.99
104	TYR	0.0	102.35	0.81
105	CYS	0.0	5.28	1.0
106	ASN	0.0	58.82	1.0

**Table 4:** Linear B-cell epitope prediction using the IEDB tool.

No.	Start	End	Peptide	Length
1	6	34	TFLKPVATENQDLKDGGFAFPPTNPLMSP	29
2	43	54	LYKDNEYVKNLD	12
3	65	85	NMNPDNDKNSNYKYPAVYDYE	21
4	99	112	NNGPRYCNKDQSKR	14
5	121	127	AKDKSFQ	7
6	137	146	VDNWEKVCPR	10

**Table 5:** Linear B-cell epitope prediction using the SVMTrip tool.

Rank	Location	Epitope	Score	Recommended ^*∗*^
1	31–48	LMSPMTLDHMRHLYKDNE	1.000	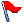
2	49–66	YVKNLDELTLCSRHAGNM	0.941	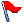
3	5–22	TTFLKPVATENQDLKDGG	0.515	
4	102–119	PRYCNKDQSKRNSMFCFR	0.471	
5	130–147	TYLSKNVVDNWEKVCPRK	0.462	
6	79–96	PAVYDYEDKKCHILYIAA	0.338	

^*∗*^The epitopes recommended are labeled by the flags.

**Table 6:** Pairwise comparison of protein sequences of Domain I of AMA1 across human *Plasmodium* species using the pairwise sequence alignment EMBOSS Needle tool.

Human *Plasmodium* species pairs	% identity	Scores
Pf/PV	54	466
Pf/Pm	50	445
Pf/Po	43	389
Pf/Pk	50	430
Pv/Pm	70	607
Pv/Po	70	607
Pv/Pk	83	693
Pm/Po	70	598
Pm/Pk	69	578
Po/Pk	72	612

Pf = *Plasmodium falciparum*; Pv = *Plasmodium vivax*; Pm = *Plasmodium malariae*; Po = *Plasmodium ovale*; Pk = *Plasmodium knowlesi*.

## Data Availability

All data generated or analysed during this study are included in this manuscript.

## Supplementary Materials

Supplementary Materials The protein sequence obtained in the study is that of Domain I of *Plasmodium falciparum* AMA1 (Figure. S1). This protein sequence is an immunogen and is one of the targets for drugs like imidazole and the sulphate ion. Using the bioinformatic program VaxiJen V2.0, the protein sequence obtained from the study was examined for immunological characteristics because not all antigens are immunogens. The outcome shows that the protein sequence is an immunogen also known as the protective antigen (Figure.S3). The sequence is therefore a promising option for a malaria vaccine. The sequence is conserved across *Plasmodium falciparum*. Different amino acid sequences of *Plasmodium* AMA1 Domain I exist. GPRYC and PAVYD, two short peptide sequences, are conserved in all *Plasmodium* species (Figure.S2). These short peptide sequences may make up *Plasmodium* AMA1's hydrophobic pocket. Evaluating the Domain I protein sequences of the other important *Plasmodium* species and the major human *Plasmodium* species, the findings show that *Plasmodium falciparum* and *Plasmodium reichenowi* are closely related and that *Plasmodium vivax* and *Plasmodium cynomolgi* are closely related (Table.S1).
