# Supplementary material for: Molecular Assessment of Domain I of Apical Membrane Antigen I Gene in Plasmodium falciparum: Implications in Plasmodium Invasion, Taxonomy, Vaccine Development, and Drug Discovery
Source: Can J Infect Dis Med Microbiol. 2022 Oct 7;2022:1419998. doi: 10.1155/2022/1419998 (PMC9568357; doi:10.1155/2022/1419998)
Supplement: Supplementary Materials — The protein sequence obtained in the study is that of Domain I of Plasmodium falciparum AMA1 (Figure. S1). This protein sequence is an immunogen and is one of the targets for drugs like imidazole and the sulphate ion. Using the bioinformatic program VaxiJen V2.0, the protein sequence obtained from the study was examined for immunological characteristics because not all antigens are immunogens. The outcome shows that the protein sequence is an immunogen also known as the protective antigen (Figure.S3). The sequence is therefore a promising option for a malaria vaccine. The sequence is conserved across Plasmodium falciparum. Different amino acid sequences of Plasmodium AMA1 Domain I exist. GPRYC and PAVYD, two short peptide sequences, are conserved in all Plasmodium species (Figure.S2). These short peptide sequences may make up Plasmodium AMA1's hydrophobic pocket. Evaluating the Domain I protein sequences of the other important Plasmodium species and the major human Plasmodium species, the findings show that Plasmodium falciparum and Plasmodium reichenowi are closely related and that Plasmodium vivax and Plasmodium cynomolgi are closely related (Table.S1). [file 1419998.f1.docx]

ENSNTTFLKPVATENQDLKDGGFAFPPTNPLMSPMTLDHMRHLYKDNEYVKNLDELTLCS RHAGNMNPDNDKNSNYKYPAVYDYEDKKCHILYIAAQENNGPRYCNKDQSKRNSMFCFRP AKDKSFQNYTYLSKNVVDNWEKVCPRKNL

**Figure.S1: Protein sequence (149 aminoacids) translated from the 459bp *Plasmodium falciparum* AMA1gene (Pf AMA1) sequence obtained in the study**

NSDVSFLKPVATGDQRLKDGGFAFPNANDHISPMTIANLKARYKDNVEMMKLNDIALCR THAASFVMAGDQNSSYRHPAVYDEKEKTCHMLYLSAQENMGPRYCSSDAQNRDAVFCFKP

DKNESFENLVYLSKNVRNDWDKKCPRKNL

Protein sequence of Domain I of *Plasmodium* vivax AMA1

ENSNVNFLTPVATGNRNLKSGGFAFPATDDHISPVTIEVLRKRYEEHADLMNLNDLSLCS KHASSFVISDDLNTSYRHPAVYDEKTKTCYILYLSAQENIGPRYCSKDAADKDTMFCFKP

AKTDNFKHYAYLSKNVVSDWDVKCPRKSL

Protein sequence of Domain I of *Plasmodium malariae* AMA1

ENSEVSFLKPVATGNEKLKSGGFAFPLTDYHISPISLQNLKRRYNENVELMKLNDMSLCA KHASSFVISEDQNTTYRHPAVYDEKEQTCYILYLSAQENLGPRYCSNDAADKDSIFCFKP EKNESFQNYVYLSKNLRDDWSSKCPRNNL

Protein sequence of Domain I of *Plasmodium ovale* AMA1

ENSKVSFLTPVATGAQRLKEGGFAFPNADDHISPITIANLKERYKENADLMKLNDIALCK

THAASFVIAEDQNTNYRHPAVYDEKEKTCYMLYLSAQENMGPRYCSPDSQNKDAMFCFKP DKNEKFDNLVYLSKNVRNDWENKCPRKNL

Protein sequence of Domain I of *Plasmodium knowlesi* AMA1

QNSEVSFLTPVATGDQSVRSGGLALPKTDVHLSPITIDNLKTMYKEHTEIVKLNNMSLCA KHTSFYVPGNNANSAYRHPAVYDKSNSTCYMLYVAAQENMGPRYCSNNANNDNQPFCFTP EKIEKYKNLSYLTKNLRDDWETSCPNKSI

Protein sequence of Domain I of *Plasmodium berghei* AMA1

ENSNTTFLTPVATGNQDLKDGGFAFPPTNPLMSPMSLDDMRNFYKDNENIKNLDELTLCS RHAGNMVPDNDKNSNYKYPAVYDEQNKKCHILYIAAQENNGPRYCNKDQSKRNSMFCFRP TKDKSFQNYTYLSKNVVDNWEKVCPRKNL

Protein sequence of Domain I of *Plasmodium reichenowi* AMA1

ENSEVSFLRPVATGDQKLKDGGFAFPNADDHISPMTIDNLKERYKDNVEMMKLNDIALCR THAASFVMAGDQNSSYRHPAVYDEKEKTCHMLYLSAQENVGPRYCNRDAENRDAMFCFKP

DKTVDFENLVYLSKNVRNDWEEKCPRKNL

Protein sequence of Domain I of *Plasmodium cynomolgi* AMA1

**Figure.S2 : Similar Domain I Protein sequences of Plasmodium species AMAI obtained from NCBI data base**

| \| Model selected: parasiteThreshold for this model: 0.5 **Your Sequence:**  ENSNTTFLKPVATENQDLKDGGFAFPPTNPL  MSPMTLDHMRHLYKDNEYVKNLDELTLCSRH  AGNMNPDNDKNSNYKYPAVYDYEDKKCHILY  IAAQENNGPRYCNKDQSKRNSMFCFRPAKDK  SFQNYTYLSKNVVDNWEKVCPRKNL    Overall Prediction for the Protective Antigen = **0.5436** ( Probable **ANTIGEN** ). \| \| --- \| |
| --- | --- |

**Figure S3: Prediction of Protective Antigen (immunogen) and Subunit vaccine sequence using Vaxijen V2.0 Bioinformatic tool**

**Table.SI: Pair wise comparison of protein sequences of Domain1 of AMA1 in major human Plasmodium species and some major Plasmodium species using Pair wise sequence alignment EMBOSS Needle tool**

| Major Human Plasmodium species and some Major Plasmodium species pair | % Identity | scores |
| --- | --- | --- |
| Pf/Pb | 43% | 389 |
| Pv/Pb | 54% | 482 |
| Pf/Pc | 54.4% | 468 |
| Pv/Pc | 89.3% | 734 |
| Pf/Pr | 91.3% | 753 |
| Pv/Pr | 55% | 478 |

Pf= *Plasmodium falciparum,* Pv= *Plasmodium* vivax, Pb= *Plasmodium berghei,* Pc= *Plasmodium cynomolgi*, Pr= *Plasmodium reichenowi*
